# S-adenosylmethionine administration inhibits levodopa-induced vascular endothelial growth factor-A expression

**DOI:** 10.18632/aging.103863

**Published:** 2020-11-07

**Authors:** Yuanliang Yan, Qijia Yan, Long Qian, Yueping Jiang, Xi Chen, Shuangshuang Zeng, Zhijie Xu, Zhicheng Gong

**Affiliations:** 1 Department of Pharmacy, Xiangya Hospital, Central South University, Hunan, China; 2 Institute for Rational and Safe Medication Practices, National Clinical Research Center for Geriatric Disorders, Xiangya Hospital, Central South University, Hunan, China; 3 Department of Pathology, Xiangya Hospital, Central South University, Hunan, China

**Keywords:** L-dopa, S-adenosylmethionine, vascular endothelial growth factor-A, acetylation, angiogenesis

## Abstract

Background: Studies have demonstrated that S-adenosylmethionine could effectively affect the clinical wearing-off phenomena of levodopa, an antiparkinsonian agent; however, the detailed mechanisms for this effect need to be further clarified.

Results: S-adenosylmethionine and levodopa had opposite effects on the protein stability of vascular endothelial growth factor-A. The analysis of tube formation and cell viability also showed the nonconforming functions of S-adenosylmethionine and levodopa on cell angiogenesis and proliferation. Meanwhile, S-adenosylmethionine could significantly abolish the increased angiogenesis and cell viability induced by levodopa. S-adenosylmethionine resulted in G1/S phase arrest, with decreased cyclin dependent kinase 4/6 and increased p16, a specific cyclin dependent kinase inhibitor. Mechanically, the different effects of levodopa and S-adenosylmethionine were dependent on the phosphorylation and activation of extracellular signal-regulated kinase. S-adenosylmethionine could be fitted into the predicted docking pocket in the crystal structure of vascular endothelial growth factor-A, enhancing its acetylation level and reducing half-life.

Conclusions: These observations suggested that methyl donor S-adenosylmethionine could act as a potential agent against vascular endothelial growth factor-A-related diseases induced by levodopa treatment.

Methods: We performed *in vitro* cytological analyses to assess whether S-adenosylmethionine intake could influence levodopa-induced vascular endothelial growth factor-A expression in human umbilical vein endothelial cells.

## INTRODUCTION

Vascular development, including vasculogenesis and angiogenesis, has been proved to be involved in several human diseases, such as neurodegenerative conditions and cancers [1]. The targeting of vascular endothelial growth factor-A (VEGFA), a crucial regulator for the pathological angiogenesis, has revealed innovative therapeutic approaches in many vascular diseases [2, 3]. Treatment with nicotinamide mononucleotide could significantly rescue the VEGF-induced angiogenic capacity in aged cerebromicrovascular endothelial cells [4]. GDF11 treatment increased VEGFA expression and secretion, leading to enhanced angiogenesis and improved neuropathological outcomes in the aged brain [5]. Furthermore, inhibition of latent membrane protein 1 by DNAzyme could suppress the microtubule-forming ability of human umbilical vein endothelial cells (HUVECs) co-cultured with nasopharyngeal carcinoma cells by downregulating VEGFA signaling pathway [6]. Thus, many studies have demonstrated the improved therapeutic effect for these diseases [7–9]; however, most of these findings are still in the pre-clinical phase. To facilitate transformation of all the therapeutic approaches for clinical application in patients, a deeper understanding of the precise modulation mechanisms of VEGFA is clearly needed in disease progression and treatment.

To date, increasing studies have demonstrated that VEGFA-positive neurons and immature angiogenesis could be frequently found in the substantia nigra of individuals with neurodegenerative conditions, indicating that angiogenic changes might be a main clinical feature of neurodegenerative pathophysiology [10]. Levodopa (L-dopa), the dopamine precursor, serves as the most effective treatment for patients with neurological disorders. However, long-term treatment with L-dopa has resulted in many VEGFA upregulation-related side-effects, such as dyskinesias. Chronic L-dopa therapy induces the overexpression of VEGFA in the corpus striatum and basal ganglia nuclei, further promoting the development of dyskinesias [11]. When co-administered with L-dopa, the VEGFA inhibitor vandetanib significantly attenuated the development of L-dopa-induced dyskinesia [12]. Meanwhile, treatment with ibuprofen or piroxicam, two cyclooxygenase inhibitors, are both proved to preserve the therapeutic effects of L-dopa by decreasing the VEGFA level with no apparent clinical hazards [11].

Epigenetic alterations, particularly DNA methylation, may play key roles in the course of nervous system injury, and could provide promising strategies for targeted therapy [13, 14]. The methyl donor S-adenosylmethionine (SAM) and DNA methyltransferase are both responsible for the maintenance of DNA methylation [15]. Under the action of catechol-O-methyltransferase, the metabolism and degradation of L-dopa requires SAM as the methyl donor. In these reactions, the methyl group released from SAM is metabolized to S-adenosylhomocysteine (SAH), which is eventually converted to total homocysteine, a well-known independent factor for increased risk of neurological disorders [16, 17]. This altered SAM/SAH ratio has been implicated to affect the clinical wearing-off phenomena and other limitations in efficacy of L-dopa during long-term treatment in patients [18]. A recent finding from Werner’s group has indicated that administration of SAM or SAM-precursors could protect the dopamine neurons against L-dopa toxicity in a catechol-O-methyltransferase-dependent manner [19].

However, whether SAM intake blocks the pro-angiogenic ability of L-dopa is still not yet interpreted clearly. To address this hypothesis, we mainly used the primary cultured and immortalized HUVECs to study the detailed roles of SAM on L-dopa-induced VEGFA expression and tube formation.

## RESULTS

### Differential effect of L-dopa and SAM on the VEGFA level in HUVECs

**Figure 1:**
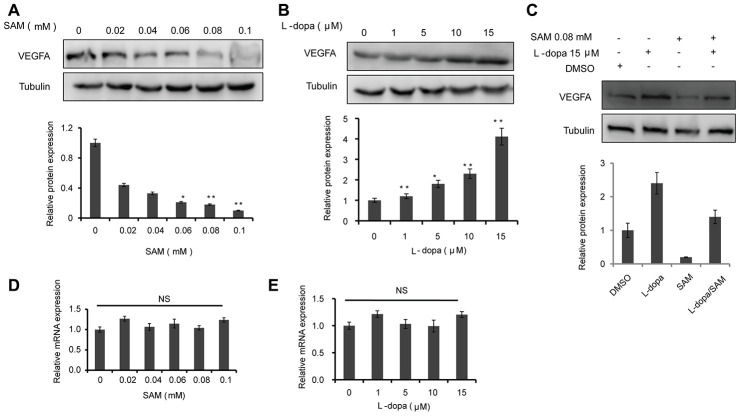
**The effects of SAM and L-dopa on the VEGFA expression in primary-HUVECs.** (**A** and **B**) Western blot analysis of VEGFA protein level in primary-HUVECs treated with indicated concentrations of SAM or L-dopa for 24 h. (**C**) Western blot analysis of VEGFA protein level in primary-HUVECs treated with combined SAM and L-dopa for 24 h. (**D** and **E**) Real-time PCR analysis of VEGFA mRNA level after different concentrations of SAM or L-dopa treatment for 24 h. α-Tubulin was used as an internal normalization control. The quantitative results shown of three independent experiments are means ± SD. The asterisk (* or **) indicates a significant (p < 0.05 or p < 0.01, respectively) compared with control groups. NS indicated no significant difference.

**Figure 2:**
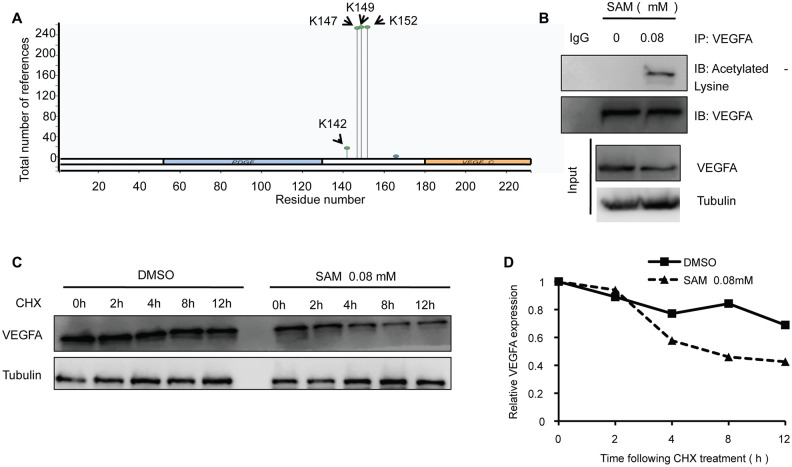
**SAM administration enhances the acetylation level of VEGFA in primary-HUVECs.** (**A**) Prediction of acetylation sites in VEGFA protein sequence by PhosphoSitePlus website. (**B**) The primary cultured HUVECs cells were treated with 0.08 mM SAM for the 24 h, followed by Co-IP with antibody against VEGFA. Immunoprecipitates were immunoblotted with the indicated antibodies. (**C**) After treated with CHX (20 μg/ml) for the indicated times, protein levels of VEGFA were determined by western blot analyses of lysates from primary-HUVECs treated with indicated concentrations of SAM. (**D**) Quantification of VEGFA protein levels relative to α-Tubulin.

To identify the effect of L-dopa and SAM on the VEGFA level, we examined the change in VEGFA expression in HUVECs using western blot and real-time PCR. The data showed that the protein levels of VEGFA are concentration-dependently downregulated in both primary cultured and immortalized HUVECs following SAM treatment, compared with the DMSO group (Figure 1A and Supplementary Figure 1A). Conversely, L-dopa treatment upregulated the VEGFA expression at the protein level (Figure 1B and Supplementary Figure 1B). Additionally, we used the non-toxic concentration of L-DOPA (15 μM) and non-toxic concentration of SAM (0.08 mM) lower than previous reported [20, 21]. Combinational treatment of L-dopa and SAM showed that SAM could weaken the L-dopa-induced VEGFA expression in primary cultured HUVECs (Figure 1C). However, neither SAM nor L-dopa could induce a significant change in VEGFA expression at the transcriptional level in HUVECs (Figure 1D, 1E and Supplementary Figure 1C, 1D). These results are different from the findings of Ohlin’s study, which demonstrated that VEGFA mRNA is upregulated following L-dopa stimulation in primary cerebral astrocytes [12]. The reason of this difference may occurred by the diversity of cell background and culture conditions. Given that posttranslational modifications (PTMs), such as acetylation [22], are important for modulating the VEGFA protein, we explored whether VEGFA is regulated in a similar manner by SAM treatment. As expected, four probable acetylation sites could be identified in full-length sequence of VEGFA protein by PhosphoSitePlus website (https://www.phosphosite.org/) (Figure 2A). Moreover, SAM significantly promoted the acetylation of VEGFA in both primary cultured and immortalized HUVECs cells (Figure 2B and Supplementary Figure 2). In addition, we performed CHX assays to further determine the protein stability of VEGFA. We found that SAM treatment could expectedly attenuate the half-life of the VEGFA protein in primary cultured HUVECs cells (Figure 2C, 2D). Collectively, these data suggest that SAM and L-dopa stimuli have opposing effects on the VEGFA protein stability in HUVECs.

### L-dopa and SAM treatment show opposing effects on the angiogenesis and proliferation of primary-HUVECs

**Figure 3:**
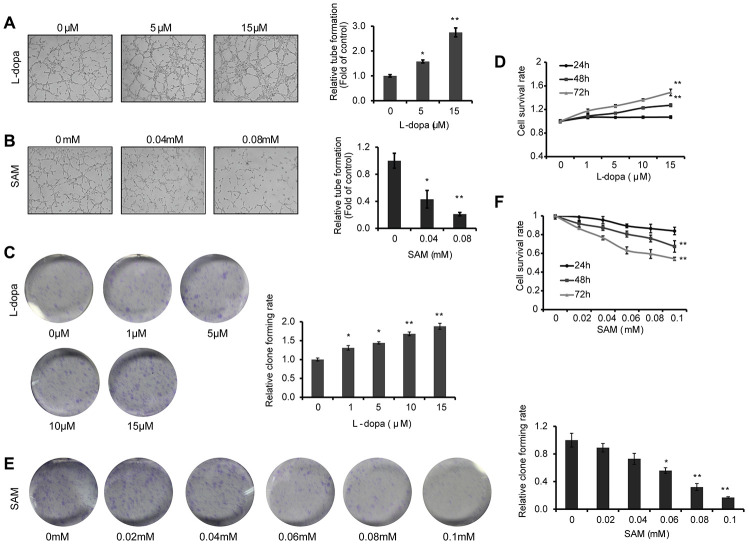
**L-dopa and SAM treatment shows opposing effects on the angiogenesis and cell proliferation.** (**A** and **B**) After treatment with the indicated concentrations of L-dopa or SAM for 24 h, endothelial tube formation was assessed using light microscopy. The average number of microtubules in 3 random horizons was analyzed using ImageJ software. (**C**) Cells were treated with L-dopa for 24 h and were incubated for another 2 weeks before fixation, staining and colony quantification. Clonogenic assays were performed in triplicate. (**D**) CCK-8 assay was using to evaluate the effect of L-dopa on the cell viability. (**E**) Colony formation assays show the effect of SAM on primary HUVECs. The quantitative results shown of three independent experiments are the mean ± SD. (**F**) CCK-8 assay was used to evaluate the effect of SAM on the cell viability. The asterisk (* or **) indicates a significant (p < 0.05 or p < 0.01, respectively) compared with control groups..

**Figure 4:**
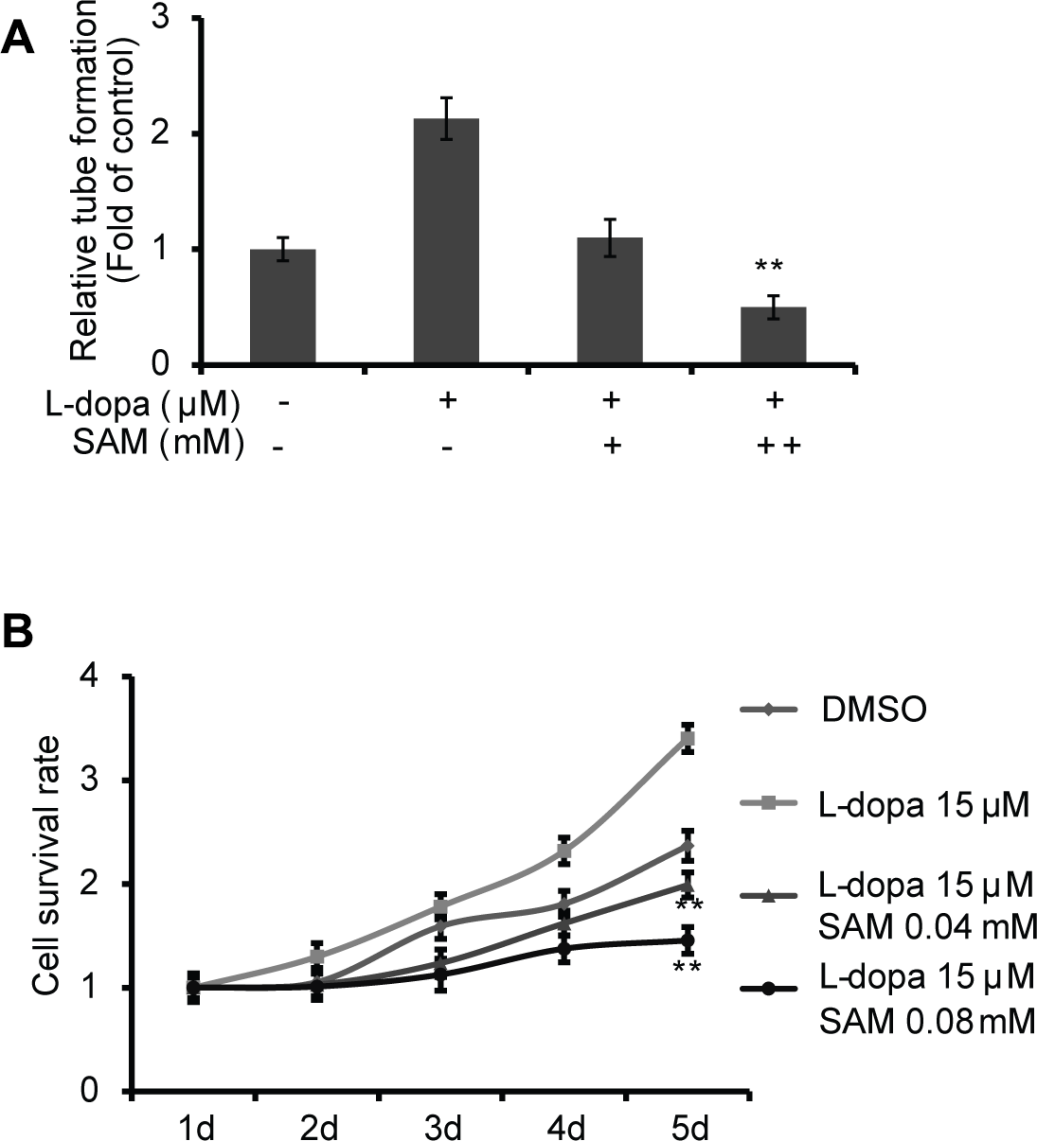
**SAM suppresses the proliferation and angiogenesis effect of L-dopa in primary-HUVECs.** (**A**) Primary HUVECs were treated with a single or combination agent for 24 h and then analyzed with tube-formation assay. The average number of microtubules from three experiments was analyzed using ImageJ software. (**B**) After treatment with the indicated conditions, cell viability was evaluated using the CCK-8 assay. The experiments were repeated for three independent times. And the quantitative results shown are means ± SD. The asterisk (* or **) indicates a significant (p < 0.05 or p < 0.01, respectively) compared with control groups.

As VEGFA is reported to play a crucial role in vascular development [23–25], we first examined whether the extent of endothelial microtubule formation is influenced by treatment with L-dopa and SAM. Compared with the DMSO group, an obvious increase of microtubule-forming ability was clearly detected in the presence of L-dopa (p<0.05) (Figure 3A). Meanwhile, SAM treatment resulted in the reduced formation of microtubules (p<0.05) (Figure 3B). Then, cell proliferation assays were performed to provide further evidence for the role of L-dopa and SAM in the regulation of cell growth. Primary HUVECs were treated with different concentrations of L-dopa and SAM for the indicated times. As shown in Figure 3C, 3D, when HUVECs were exposed to L-dopa, a significant increase in cell clone formation and viability (48h: p<0.01; 72h: p<0.01) was observed compared to that observed for DMSO treated cells. However, a strong inhibition of growth occurred upon treatment with SAM (48h: p<0.01; 72h: p<0.01) (Figure 3E, 3F). The typical images of cell colony formation from different treatments are shown in Figure 3C and 3E. In addition, we examined the combination effect of L-dopa and SAM on cell angiogenesis and proliferation in the primary-HUVECs. As expected, compared with the DMSO group, SAM could significantly abolish the L-dopa-induced promotion of tube formation ability and cell viability (0.04 mM: p<0.01; 0.08 mM: p<0.01) in a concentration-dependent manner (Figure 4A, 4B), indicating that SAM administration could block the L-dopa-mediated pro-angiogenic ability. These data all together show that treatment with L-dopa and SAM could result in opposing actions on cell angiogenesis and proliferation.

### ERK activation is involved in the effects of L-dopa and SAM

**Figure 5:**
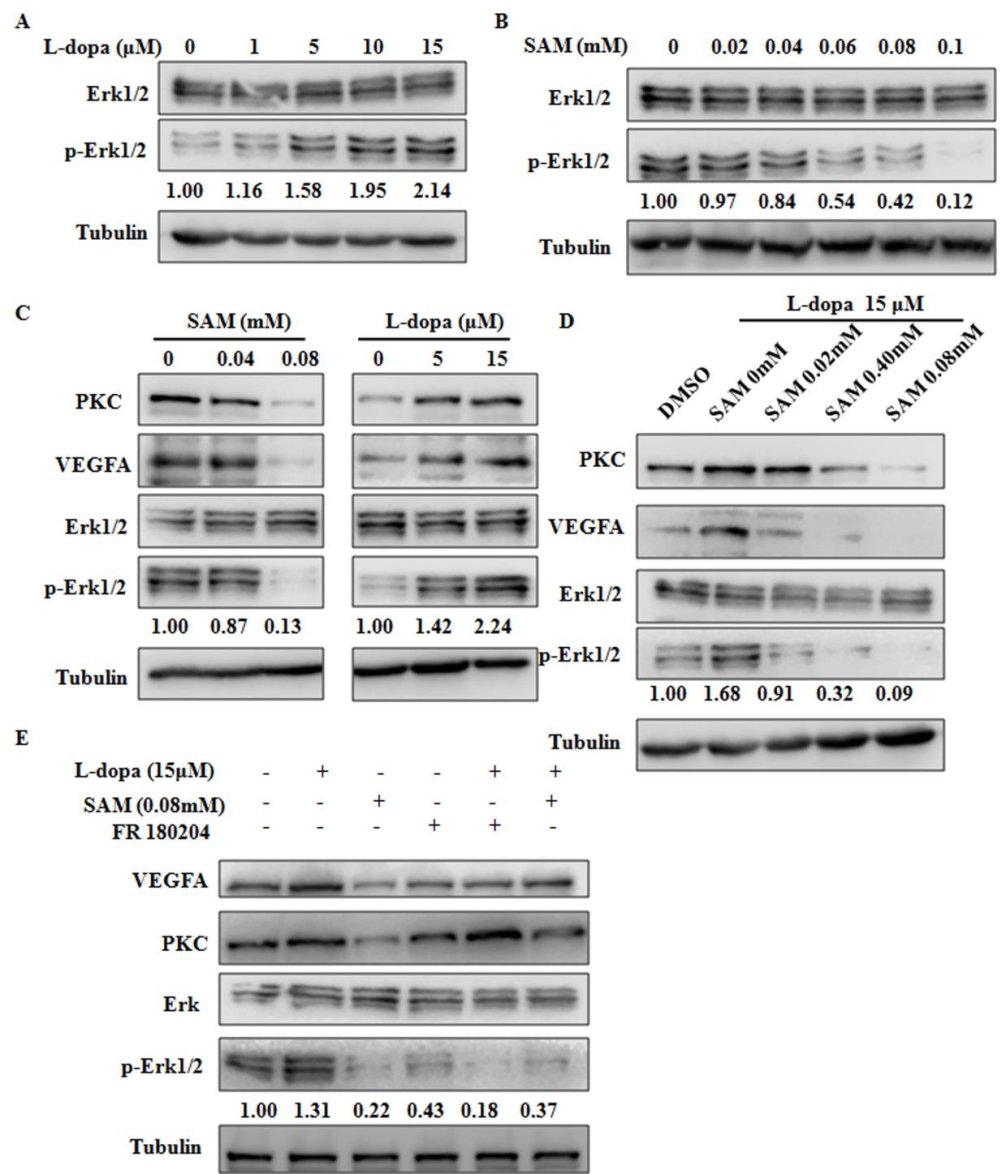
**The functions of L-dopa and SAM are dependent on the ERK activation.** (**A**–**E**) Primary HUVECs were treated by different concentrations of L-dopa and SAM and 10 μM FR180204 for 24 h. Then, the protein expression levels were analyzed by western blot with the indicated antibodies. α-Tubulin used as an internal normalization control.

Recent studies have revealed that the VEGFA-PKC-ERK signaling axis plays a pivotal role in angiogenesis and cell growth, providing a potential therapeutic strategy for various angiogenesis-dependent diseases [26]. Moreover, VEGFA-induced ERK phosphorylation at residues Thr202/Tyr204 has been shown to be strongly dependent on protein kinase C (PKC) [27, 28]. Thus, we next examined the effects of L-dopa and SAM on ERK activation. The results show that in primary cultured HUVECs, compared with the DMSO treated group, elevated levels of Thr202/Tyr204 phosphorylated ERK could be seen upon concentration-dependent L-dopa treatment, whereas the inverse change was seen after SAM intake (Figure 5A, 5B). Similar trend changes of PKC level could also be clearly noticed upon L-dopa and SAM treatment (Figure 5C). Furthermore, SAM abrogated the upregulation of VEGFA-PKC-ERK signaling induced by L-dopa in a concentration-dependent manner (Figure 5D). To further investigate the role of ERK signaling in the L-dopa response, we inhibited ERK activity with adenosine triphosphate-competitive ERK1/2 inhibitor FR180204 [29]. As expected, ERK inhibitor FR180204 yielded similar results to SAM on the L-dopa response. Pharmacologic inhibition of ERK activation by FR180204 abolished the L-dopa-induced upregulation of the VEGFA protein level. In addition, no significant change was seen in the expression of PKC after ERK inhibition by FR180204 (Figure 5E), supporting that PKC is an upstream regulator for ERK activation [30]. These results collectively indicated that the L-dopa-mediated VEGFA upregulation is dependent of ERK phosphorylation and activation.

### SAM intake leads to the cell-cycle arrest at the G1/S phase

**Figure 6:**
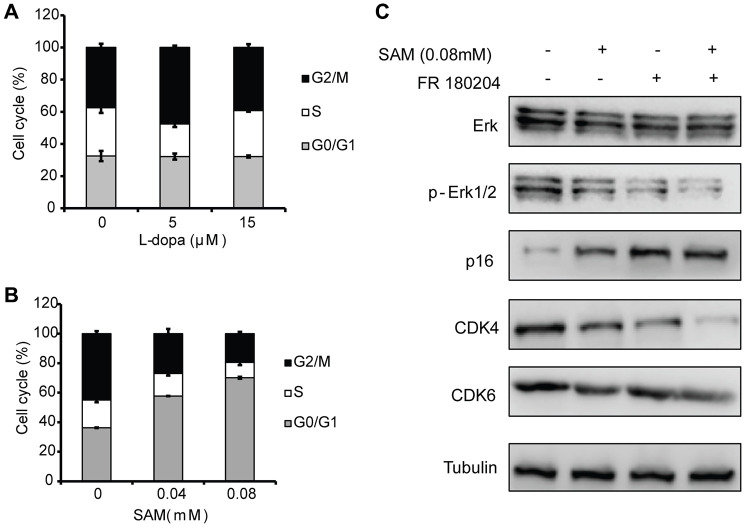
**Effects of SAM on the cell cycle distribution in Primary-HUVECs.** (**A**) Upon treatment with SAM at 0.04 mM and 0.08 mM for 24 h, the cell-cycle progression of primary HUVECs was analyzed by flow cytometry. (**B**) Flow cytometry was used to assess the cell cycle in primary HUVECs treated by L-dopa at 5 μM and 15 μM for 24 h. (**C**) Cell cycle-related proteins (CDK4/6 and p16) were determined using western blot analysis. α-Tubulin was used for the loading control. The quantitative results shown of three independent experiments are means ± SD.

Since cell proliferation could be effectively regulated by cell cycle progression [31], we next examined the effect of L-dopa and SAM on cell cycle distribution. Different concentrations of L-dopa and SAM were administered to primary HUVECs after overnight serum starvation, and cell cycle distribution was assessed using the flow cytometry method. Compared with the DMSO treated group, L-dopa treatment could lead to an increase in the number of proliferative cells (Figure 3C, 3D), but no obvious changes in cell cycle exists (Figure 6A). The reasons for the pro-proliferation effect of L-dopa might be due to other factors, such as the prolonged mitosis [32], and not the cell cycle arrest. However, SAM significantly increased the percentage of cells in the G0/G1 phase (57.69 ± 0.30 for 0.04 mM SAM, 70.11 ± 1.40 for 0.08 mM SAM) compared to DMSO-treated cells (36.25 ± 1.90). This increase was coupled with a significant decrease in the percentage of cells in the G2/M phase (26.93 ± 1.30 for 0.04 mM SAM, 19.54 ± 1.22 for 0.08 mM SAM) (Figure 6B). It is well known that cyclin-dependent kinases (CDKs) function as the key factor that drives cell cycle progression [33]. To further confirm the G1/S phase arrest phenotype due to SAM intake, we investigated the cell cycle-associated checkpoint CDK4/6, the regulators required for G1/S transition [34]. As shown in Figure 6C, it was found that SAM markedly decreased the CDK4/6 expression level while increasing the level of p16, a specific CDK inhibitor [35]. Moreover, the combination of SAM and FR180204 could further suppress the G1 phase regulators CDK4/6, which further indicates the important roles of ERK signaling in the effect of SAM in HUVECs. Taken together, these findings demonstrate that SAM has a preferential pro-cell cycle arrest effect on the primary HUVECs.

### Molecular docking analysis

**Figure 7:**
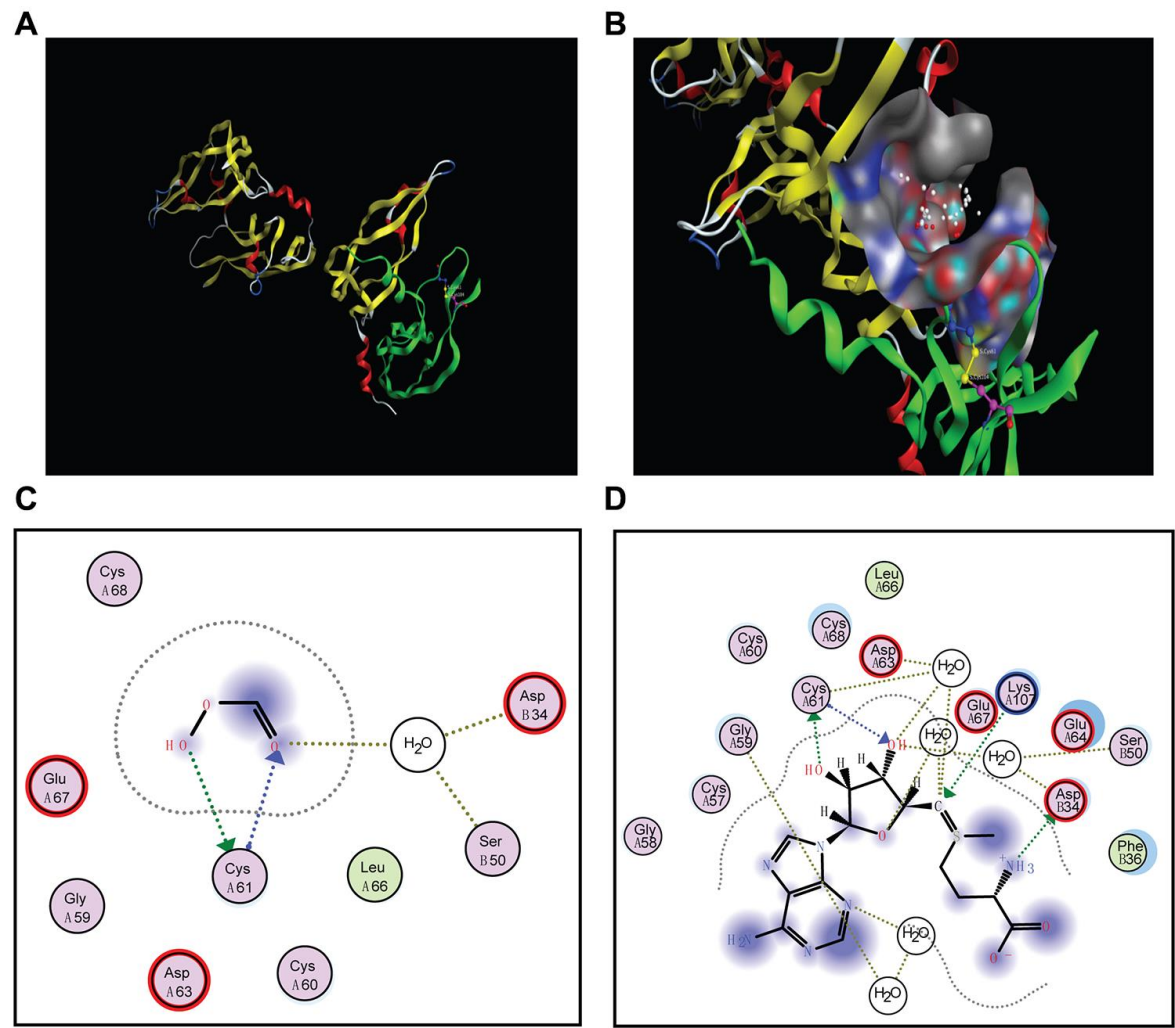
**Docking poses and activity pocket for SAM in the crystal structure of VEGFA (PBD ID: 1MKK).** (**A**) The disulfide bridges between C61A-C104A of VEGFA. (**B**) Docking site of VEGFA crystal structure. (**C** and **D**) Important residues are labeled and shown as sticks to facilitate the localization of PFA or SAM on the active site region in VEGFA.

The MOE software was used to analyze the docking poses and binding energies between SAM and VEGFA to evaluate the possible activity pocket for SAM in the VEGFA protein. A recent study clarified that VEGFA is a cystine knot growth factor, which consists of three highly intertwined disulfide bridges. The cystine knot motif is a major determinant for the thermodynamic stability of VEGFA because it interlocks four separate chain segments (Figure 7A) [36]. In addition, treatment with PFA oxidation could remove the intermolecular disulfide bridge, leading to an unstable structure of the protein [37]. By studying the crystal structural properties of the mutant Cys61Ala-Cys104Ala, Muller et al. found that the disulfide bridges in Cys61Ala-Cys104Ala are crucial for the thermal stability and structural function of VEGFA [38]. Therefore, we mainly focused on whether SAM could interact with the Cys61Ala-Cys104Ala domain of VEGFA. After the substrate pocket was predicted in the Cys61Ala-Cys104Ala domain of VEGFA (Figure 7B), we found PFA oxidation was docked into the pocket as expected, with an E-score^2^ of -2.967 to -3.112 (Figure 7C, Supplementary Figure 3A), indicating that the oxidation of PFA could attenuate the stability of VEGFA directly. As illustrated in Figure 7D and Supplementary Figure 3B, SAM fit the same active pocket in VEGFA as PFA, including amino acids Cys61, Lys107 and Asp34. In addition, the E-score^2^ values of the docking parameter for SAM (-4.834~ -5.517) are lower than those of PFA. Taken together, these results show good affinity of SAM with VEGFA by forming several potent hydrogen bonds between their polar moieties and the amino acid residues.

## DISCUSSION

**Figure 8:**
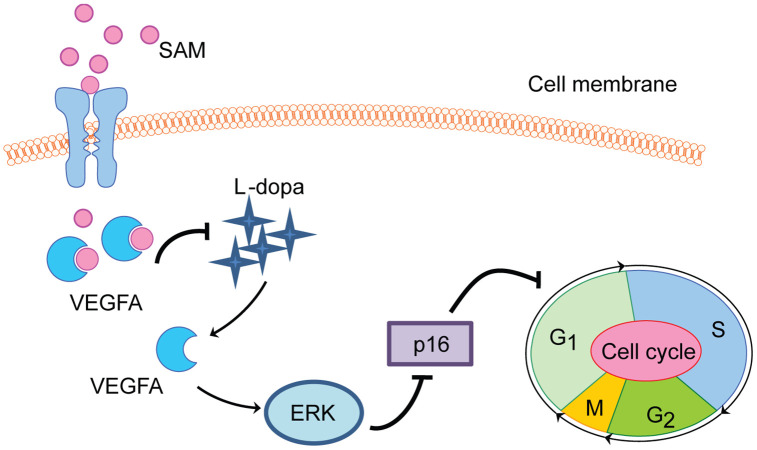
**Proposed schematic of SAM effect on L-dopa-induced angiogenesis and proliferation in HUVEC cell.** SAM intake significantly suppresses the L-dopa-induced VEGFA upregulation. Different from L-dopa treatment, SAM could promote the cell cycle arrest at G1/S phase arrest, accompanied with the decreased tube formation ability and cell proliferation rate. In addition, the data from molecular docking analysis indicates the direct binding ability between SAM and VEGFA.

The purpose of this investigation was to identify the modulation mechanism of SAM on L-dopa-induced VEGF expression. In the current study, using the cultured HUVEC model and cell biology techniques, we present potential insight into the functions of SAM on L-dopa-induced VEGFA expression and tube formation. In the first step, we discovered the opposite effects of L-dopa and SAM on the VEGFA protein level and cell proliferation rate. Then, molecular mechanism studies revealed that SAM administration, blocking the pro-angiogenic ability of L-dopa, is dependent on the ERK activation to some extent (Figure 8).

Recently, SAM has been used for the treatment of several human diseases, especially neurodegenerative conditions. Preliminary evidence suggests that SAM treatment ameliorated symptoms in certain neurocognitive and psychotic disorders, indicating that SAM holds promise as an alternative strategy for multiple neuropsychiatric conditions [39, 40]. Acting as an antioxidant, SAM could effectively protect nerve cells against amyloid-β-induced cellular injury by inhibiting oxidative stress and neuroinflammation in Alzheimer's disease [41]. However, intracerebroventricular injection of SAM induced the symptoms of Parkinson's disease by destroying the methylation/demethylation homeostasis of prenylated proteins [42]. Due to the limited evidence, these unexpected and confused findings about SAM for the treatment of neurological disorders should be evaluated in the future. In addition, long-term administration of SAM also showed potential antitumor effects in human cancer [21, 43]. SAM administration promoted VEGFC promoter methylation and downregulated its expression in gastric cancer cells [44].

It is known that VEGFA is upregulated in patients with neurodegenerative disorders after chronic L-dopa therapy, leading to some pathological angiogenesis associated diseases such as symptoms of motor dysfunction. That is because high concentrations of VEGFA result in the formation of abnormal blood vessels and disruption of the blood brain barrier, which are proved to be causative factors for the degeneration of dopaminergic neurons [45]. Treatment with VEGFA-targeted drugs could effectively preserve the curative effect of L-dopa while delaying the development of dyskinesia [11]. Additionally, L-dopa therapy in patients depletes the cellular concentrations of the methyl donor, SAM, and thus results in a reduced SAM/SAH ratio [46]. SAM administration has been proved to influence the wearing-off phenomena and other clinical problems of L-dopa during long-term treatment [18]. Accordingly, we found that L-dopa can significantly promote the VEGFA expression at the protein level and accelerate angiogenesis in HUVECs. However, the upregulated VEGFA level and tube-forming ability are both impaired by SAM in a concentration-dependent manner. Moreover, we clarified that SAM could be docked into the predicted pocket of VEGFA, further promoting the VEGFA acetylation and attenuating its half-life, indicating that SAM could effectively reduce L-dopa-induced VEGFA protein stability through direct molecule binding.

Nowadays, increasing reports have demonstrated that the expression and activity of VEGFA could be regulated at the post-translational level. The polyADP ribosylation and glycosylation are the two major post-translational modifications of VEGFA, which influence its secretion and biological function [47]. In addition, histone H3 mediated VEGFA acetylation promoted VEGFA upregulation in injured peripheral nerves, thus participating in angiogenesis and reinforcing pain behaviors [48]. The previous studies showed that phosphorylation modification of VEGFA plays important roles in VEGFA regulated angiogenesis in HUVECs [49]. Our present study demonstrated L-dopa elevated levels of ERK phosphorylation whereas the inverse change was seen with SAM treatment. As SAM can promote the acetylation of VEGFA, further research is needed to clarify the effect of SAM on the phosphorylation of VEGFA.

Another interesting finding is that the changes of VEGFA level and cell proliferation rate by L-dopa may be due to their modulation upon ERK activation. As previously mentioned, the signal transduction cascade, the ERK signaling pathway, was closely correlated with the concentration of L-dopa and is frequently altered following chronic L-dopa treatment [50]. Using western blot assays, we found that FR180204, an ERK activation inhibitor, could relieve the efficacy of L-dopa-induced upregulation of VEGFA. At the same time, downregulation of the ERK signaling pathway by FR180204 results in an enhanced inhibitory effect of SAM on the cell-cycle checkpoint proteins CDK4/6 in primary HUVEC cell lines. Taken together, EKR inhibitors may hypothetically contribute to decrease the severe negative effects during long-term treatment with L-dopa in patients with Parkinson's disease.

This study, however, is subject to several limitations. First of all, the effect evaluated in the model was mainly based on the data from the primary cultured and immortalized HUVECs *in vitro*. These findings might be therefore subjected to some biases. In the future, the biological functions of SAM-VEGF signaling axis should be strengthened in more comprehensive researches, such as animal models and clinical trials. Second, we were unable to show the detailed mechanisms for VEGFA acetylation modification. All these factors need to be further clarified to understand their dynamics and functional implications. In conclusion, the objective of this report was to explore the roles of SAM on VEGF modulation. This study confirms meaningful clues that a temporary SAM intake can ameliorate L-dopa-induced VEGFA upregulation-associated disorders.

## MATERIALS AND METHODS

### Cell lines and reagents

With signed informed consent forms and following ethical standards from the research ethics committee, Xiangya Hospital, China (No. 201402007), umbilical cords were obtained from randomly selected healthy mothers after they gave birth, and stored at 2-8°C. Then, primary cultures of HUVEC were on-set after enzymatic digestion of endothelial tissue from umbilical cord veins, as previously reported [51]. In brief, HUVECs were isolated with dispase solution (Gibco, Grand Island, USA), pre-warmed at 37°C within 1 to 4 h after birth. The obtained HUVECs were then propagated in cell culture dishes with EGM-2 Endothelial Cell Growth Medium-2 (Lonza, MD, USA) supplemented with 10% fetal bovine serum (FBS, HyClone, UT, USA). Immortalized HUVECs (ATCC CRL-1730) were purchased from Xiangya Central Experiment Laboratory (Hunan, China) and were routinely cultured in RPMI Medium 1640 (Gibco, Grand Island, USA) supplemented with 10% FBS. L-dopa (V900425, Sigma) and FR180204 (S7524, Selleck Chemicals) were dissolved in 0.1% dimethylsulfoxide (DMSO). In addition, SAM (A506555) was purchased from Sangon Biotech (China) and cycloheximide (CHX, 2112) was purchased from Cell Signaling Technology. The respective concentrations used can be seen in the results.

### Western blot and Co-immunoprecipitation (Co-IP)

Briefly, approximately 1 × 10^6^ endothelial cells were seeded onto 6-well plates and cultured with the indicated concentrations of L-dopa or SAM for 48 h. The antibodies for western blot analysis were as follows: anti-α-Tubulin (69969, Santa Cruz), anti-VEGFA (57496, Santa Cruz), anti-acetylated lysine (9441, Cell Signaling Technology), anti-extracellular signal-regulated kinase (ERK) (ab54230, Abcam), anti-phospho-ERK (ab65142, Abcam), anti-protein kinase C (PKC) (17804, Santa Cruz), anti-p16 (468, Santa Cruz), anti-CDK4 (12790, Cell Signaling Technology), and anti-CDK6 (13331, Cell Signaling Technology). Protein expression levels were determined by western blot technology as previously described [52]. In brief, cell extracts were prepared in cold Pierce IP buffer (Thermo Scientific, 87788), and then subjected to 10% SDS-PAGE and transferred to polyvinylidene fluoride membranes. The membranes were blocked with 5% fat-free milk and incubated with the indicated antibodies. At last, the immunoreactive bands were visualized with SuperSignal^®^ West Femto Maximum Sensitivity Substrate (Thermo Scientific, 34095). For Co-IP, cell lysates were clarified by immunomagnetic separation and incubated with the indicated antibody plus Dynabeads^®^ Protein A (Thermo Scientific, 10002D) at 4 °C. After extensive washing with lysis buffer, the immunocomplexes were analyzed by western blot as described.

### Real-time PCR

Total RNA was isolated from the HUVECs and followed by cDNA synthesis as previously described [53]. Real-time PCR was performed with Power SYBR Green PCR Master Mix (Applied Biosystems, 4367659) using an ABI 7500 instrument. The primers for detecting VEGFA and α-Tubulin were shown in Supplementary Table 1.

### Tube formation assay

The tube formation test was analyzed to evaluate the effect of L-dopa and SAM on angiogenesis using a previously published process [6, 54]. Briefly, we first treated HUVEC cells with different concentrations of SAM or L-dopa, and 24 h later, cells were trypsinized and centrifuged at 600 × g for 5 min. Then approximately 5 × 10^4^ cells were seeded into 24-well plates with 200 μl of embedded Matrigel (BD Biosciences, NJ, USA). Then, the cells were incubated for 12 h, and the extent of endothelial microtubule-forming was measured using a light microscope.

### Cell viability assay

After treated with the indicated conditions, approximately 1 × 10^3^ primary HUVECs were seeded into 96-well plates and then incubated for 0 h, 24 h, 48 h, 72 h, etc. Then, 10 μl CCK-8 reagent was added to each well, and then cell viability was evaluated using a spectrometer at 450 nm according to the instructions provided (Selleck Chemicals, USA).

### Clonogenic survival assay

The HUVECs (approximately 2 × 10^3^) were seeded into 6-well plates and incubated for 24 h. The cells were then treated with different concentrations of L-dopa or SAM. After approximately 15 days, cells were washed with PBS, fixed in ice-cold methanol and stained with crystal violet. The cell clones were counted using ImageJ 1.52v software (National Institutes of Health, MD, USA), and survival curves were quantified using Microsoft Excel 2007 (Microsoft Office system, USA)

### Flow cytometric analysis

Flow cytometry was performed to evaluate the cell cycle distribution of HUVECs treated with L-dopa or SAM using a published method [55]. Briefly, approximately 1 × 10^6^ endothelial cells were seeded onto 6-well plates and cultured with the indicated concentrations of L-dopa or SAM for 24 h. All cells were washed with ice-cold PBS, fixed in 70% ethanol, and then stored at 4°C overnight. After washing with PBS again, the fixed cells were stained with 0.1% RNase A and 50 μg/ml propidium iodide at 25°C for approximately 30 min in a dark place and then assayed with guava easyCyte 12HT Benchtop Flow Cytometer (Millipore, Bedford, USA). Finally, the cell cycle parameters were determined using the CellQuest software program (Version 5.1, Becton, Dickinson and Company, USA).

### Molecular modeling

To investigate the possible binding sites of SAM in the VEGFA protein, a molecular docking analysis was conducted using MOE v2014.090 software (Chemical Computing Group). The crystal structure of VEGFA (PBD ID: 1MKK) was downloaded from the RCSB Protein Data Bank [56] (http://www.rcsb.org/pdb/explore/explore.do?structureId=1mkk). In addition, the 2D or 3D structures of SAM were both obtained from the PubChem website [57] (https://pubchem.ncbi.nlm.nih.gov/). Regularization and optimization for VEGFA and SAM were performed with internal default parameters. In the analysis process, the active site of VEGFA was considered a rigid molecule, whereas the phytochemicals were treated as being flexible. The docked performic acid (PFA) was assigned a score according to its fit in the ligand binding pocket of VEGFA, serving as the control binding mode.

### Statistical analysis

Data are presented as the mean ± standard deviation (SD). Statistical comparisons of data were performed with Student’s t-test and one-way ANOVA using the SPSS15.0 software (Chicago, IL, USA). p value < 0.05 was considered statistically significant.

### Ethical approval

This article does not include any studies with human participants or animals. The research ethics committee of Xiangya Hospital approved this study (201402008), and that it was conducted in accordance with the Declaration of Helsinki.

## Supplementary Material

Supplementary Figures

Supplementary Table 1
