# Supplementary material for: S-adenosylmethionine administration inhibits levodopa-induced vascular endothelial growth factor-A expression
Source: Aging (Albany NY). 2020 Nov 7;12(21):21290–307. doi: 10.18632/aging.103863 (PMC7695432; doi:10.18632/aging.103863)
Supplement: Supplementary Table 1 [file aging-12-103863-s002..pdf]

SUPPLEMENTARY FIGURES

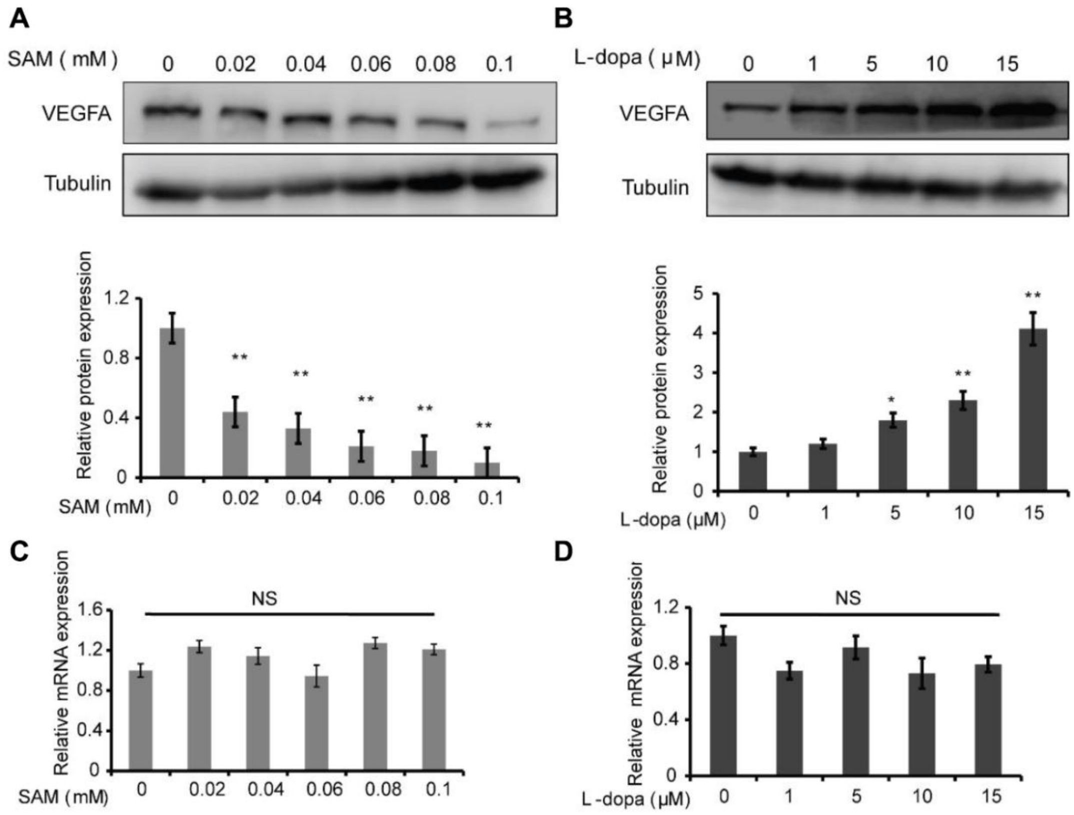

Supplementary Figure 1. The effects of SAM and L-dopa on the VEGFA expression in immortalized HUVECs. (**A** and **B**) After treatment with indicated concentrations of SAM or L-dopa for 24 h, western blot was used to determine the VEGFA protein level in immortalized-HUVECs. (**C** and **D**) Real-time PCR analysis of VEGFA mRNA level after different concentrations of SAM or L-dopa treatment for 24 h in immortalized-HUVECs. α-Tubulin was used for the loading control. The experiments were repeated for three independent times. And the quantitative results shown are means ± SD. * and ** indicated the significant difference. NS indicates no significant difference.

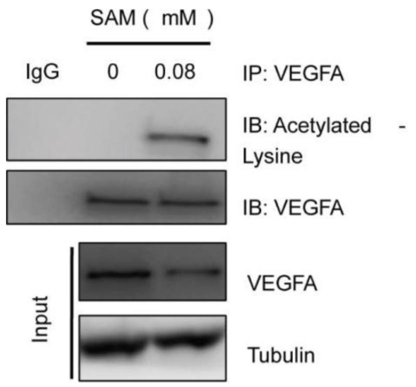

Supplementary Figure 2. SAM administration enhances the acetylation level of VEGFA in immortalized HUVECs. The immortalized HUVECs cells were treated with 0.08 mM SAM for the 24 h, followed by Co-IP with antibody against VEGFA. Immunoprecipitants were immunoblotted with the indicated antibodies.

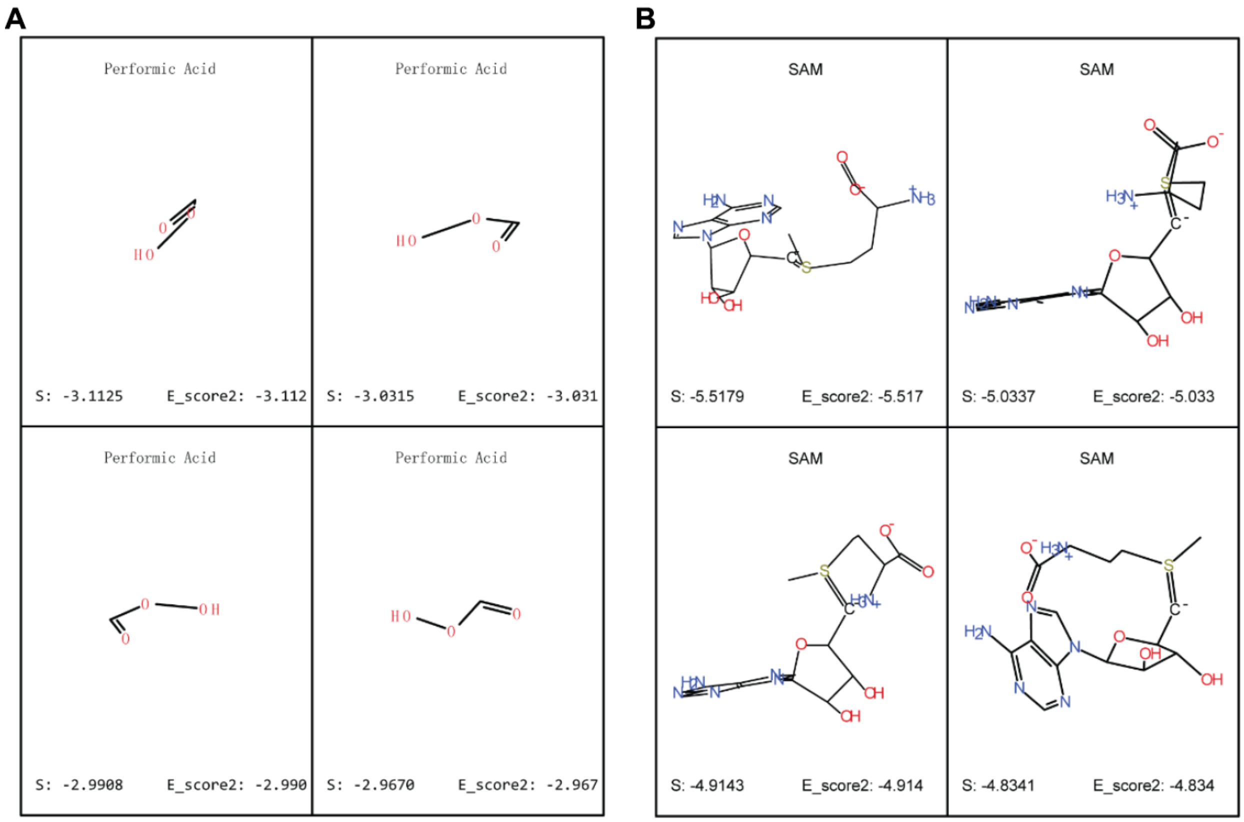

Supplementary Figure 3. The docked energy value between SAM and VEGFA. (**A** and **B**) As predicted, SAM fit the active pocket in VEGFA with the E-score[2] values of -4.834 to -5.517, which is less than that of control binding mode, docked performic acid.
